# Effectiveness and Safety of Different Rivaroxaban Dosage Regimens in Patients with Non-Valvular Atrial Fibrillation: A Nationwide, Population-Based Cohort Study

**DOI:** 10.1038/s41598-018-21884-y

**Published:** 2018-02-22

**Authors:** Hsin-Yi Huang, Shin-Yi Lin, Shou-Hsia Cheng, Chi-Chuan Wang

**Affiliations:** 1 Graduate Institute of Clinical Pharmacy, College of Pharmacy, National Taiwan University, Taipei, Taiwan; 2 Department of Pharmacy, Shuang Ho Hospital, Taipei Medical University, Taipei, Taiwan; 3 School of Pharmacy, National Taiwan University, Taipei, Taiwan; 4 Department of Pharmacy, National Taiwan University Hospital, Taipei, Taiwan; 5 Institute of Health Policy and Management, College of Public Health, National Taiwan University, Taipei, Taiwan

## Abstract

The objective of this study is to evaluate the effectiveness of different rivaroxaban dosage regimens in preventing ischemic stroke and systemic thromboembolism among Asians. A retrospective cohort study was conducted on data from nationwide insurance claims in Taiwan. Patients with non-valvular atrial fibrillation under warfarin or rivaroxaban therapy were included. Propensity score matching was used to balance the covariates, and Cox-proportional hazard models were applied to compare the effectiveness and safety of each treatment group. Rivaroxaban was associated with a significantly lower risk of venous thromboembolism (hazard ratio [HR]: 0.51; 95% confidence interval [CI]: 0.29–0.92, P = 0.02) and intracranial hemorrhage (HR: 0.48; 95% CI: 0.32–0.72, P < 0.001) than warfarin. Rivaroxaban 20 mg and 15 mg were associated with a significantly lower risk of ischemic stroke (20 mg, HR: 0.48; CI: 0.29–0.80, P = 0.005; 15 mg, HR: 0.69; CI: 0.53–0.90, P = 0.005), but rivaroxaban 10 mg was not. In the subgroup analysis of patients older than 65 years, the results were generally the same, except that rivaroxaban had a significantly lower risk of ischemic stroke than warfarin.

## Introduction

Atrial fibrillation (AF), the most common type of arrhythmia, increases the risk of ischemic stroke and systemic thromboembolism five-fold and contributes to 15% of all ischemic stroke cases^1,2^. Appropriate anticoagulation therapy can effectively lower the risk of thromboembolism^3^. For decades, vitamin K antagonists were the only option of oral anticoagulant treatment. However, the narrow therapeutic index and multiple drug-drug and drug-food interactions of warfarin complicate its use^4^. The launch of non-vitamin K antagonist oral anticoagulants (NOACs) is a landmark in preventing ischemic stroke among AF patients. Rivaroxaban, a factor Xa inhibitor, has been shown to be associated with a comparable risk of ischemic stroke and systemic thromboembolism and a lower risk of intracranial hemorrhage (ICH) than warfarin^5^.

Even though the efficacy and safety of rivaroxaban have been proven, data for the Asian population is inadequate, especially for different dose levels. According to the Rivaroxaban Once Daily Oral Direct Factor Xa Inhibition Compared with Vitamin K Antagonism for Prevention of Stroke and Embolism Trial in Atrial Fibrillation for Japanese (J-ROCKET AF) study, rivaroxaban 15 mg was as effective as warfarin in preventing ischemic stroke and systemic thromboembolism^6^. On the other hand, the subgroup analysis of the East Asian population in the global ROCKET AF study which used rivaroxaban 20 mg did not show any difference in efficacy and safety^7^. Therefore, the optimal regimen of rivaroxaban therapy for Asians is still under discussion.

In addition to trials, several observational studies have investigated the effectiveness and safety of NOACs in clinical use among non-valvular AF patients^8–10^, but to our knowledge, the real-world data for rivaroxaban, with a specific focus on different dosages, are sparse. This issue is particularly important because Asians have several demographic characteristics that differ from other ethnic groups. For example, Asians have a lower prevalence of AF but carry a specific increased risk of ischemic stroke, are more sensitive to warfarin, and more prone to suffer from warfarin-related bleeding. All these characteristics may lead to a change in the effectiveness and safety profile of anticoagulant therapy^7,11–17^. Thus, we aimed to conduct a nationwide, population-based study by using the National Health Insurance (NHI) claims database in Taiwan to investigate the effectiveness and safety of rivaroxaban at different dose levels in real-world practice, with a specific focus on different dosing regimens.

## Methods

### Data source

This study was conducted using claims data from the NHI program that includes more than 23 million enrollees, about 99.9% of the population in Taiwan. Comprehensive information about outpatients, inpatients, prescriptions, and insurance enrolment is included in the claims database. Data from between 2010 and 2015 were used for this study. The Research Ethics Committee of National Taiwan University Hospital approved the study.

### Study Design and Patients

This was a retrospective cohort study with a new-user design. Patients who were at least 20 years old; had at least 1 inpatient or 2 separate outpatient diagnoses of AF, identified according to the International Classification of Diseases, Ninth Revision, and Clinical Modification (ICD-9-CM) code 427.31; and were prescribed rivaroxaban or warfarin from June 1, 2012 to December 31, 2015, fulfilled the inclusion criteria. Patients were excluded if they had a prosthetic heart valve or mitral valve disease during the study period^18^, were pregnant, diagnosed with cancer, or under chronic dialysis within 12 months prior to the index date. The index date was defined as the date of the first anticoagulant prescription observed on or after June 1, 2012, the date that rivaroxaban treatment began to be reimbursed in the NHI program. Chronic dialysis was defined as 1) having at least two separate outpatient procedure codes indicating dialysis treatment was undergone for more than 90 days, or 2) being enrolled in the “registry for patients with catastrophic illness” of the NHI program because of chronic renal dialysis^19,20^.

Two study groups were obtained in our study, rivaroxaban users and warfarin users. Only patients newly prescribed with rivaroxaban or warfarin were included for analysis. Patients were considered as newly on the study drugs if they did not receive any prescriptions for the study drugs for at least 12 months before the index date. A rivaroxaban user was determined by the very first rivaroxaban prescription after June 1, 2012. Warfarin users were those who received a warfarin prescription from June 1, 2012, to December 31, 2015, without receiving a prescription for NOAC during the study period.

### Outcome Measures

Clinical effectiveness and safety were assessed separately in the follow-up period. Effectiveness was defined as the occurrence of ischemic stroke, venous thromboembolism (VTE), or transient ischemic attack (TIA). Safety was defined as the occurrence of ICH, gastrointestinal (GI) bleeding, or other bleeding. Primary diagnoses during hospitalization were applied for the assessment of the study outcomes. Since patients diagnosed with TIA may not require hospitalization, the primary diagnoses during emergency room visits were also included to detect TIA. Other bleeding was defined as patients that visited the emergency room or required hospitalization due to hemoptysis, epistaxis, hematuria, hemarthrosis, or hemopericardium. We identified ischemic stroke by ICD-9-CM codes (ICD-9-CM: 433, 434), which were validated by health insurance data in Taiwan^21^.

Patients were followed from the index date to whichever of the following events came first: 1) occurrence of the outcome of interest, 2) switching to an alternative oral anticoagulant, 3) discontinuation of the index anticoagulant, or 4) the end of the observation period (December 31, 2015). Medication discontinuation was defined as either discontinuing oral anticoagulation therapy or having a > 30-day gap between the end of an oral anticoagulant prescription and the next prescription^22,23^.

### Baseline Characteristics and Covariates

The covariates adjusted were those factors known to affect anticoagulant treatment and study outcomes, including age, gender, annual stroke risk, specific comorbidities, and concomitant medications^24–26^. Comorbidities were identified by diagnoses made within 12 months before the index date, the date when the first anticoagulant was prescribed. Concomitant medications were identified by at least one prescription within 90 days preceding the index date^24,25^.

The baseline risk of ischemic stroke was assessed by using the CHA_2_DS_2_-VASc scoring system. The calculated score was categorized into low risk (0 points), moderate risk (1 point), and high risk (≥2 points). In contrast, the baseline bleeding risk was assessed by using the HAS-BLED scoring system.

### Statistical Analysis

One-to-one propensity score matching using a greedy matching algorithm was applied to balance the covariates between the comparison groups. Absolute standardized mean differences were applied to compare the between-group balance of the baseline characteristics. An absolute standardized difference of less than 0.1 was recognized as indicating no significant difference. Cox-proportional hazard models were applied to determine the relationship between anticoagulant treatment and the study outcomes. The effectiveness and safety of rivaroxaban were first compared to warfarin in the main analysis. Subgroup analyses were performed in different dosage groups (e.g., rivaroxaban 10 mg, 15 mg, and 20 mg), and age groups (e.g., ≥65 years and ≥80 years). Two-sided tests with an α < 0.05 were defined as statistically significant. All statistical analyses were performed with SAS software version 9.4 (SAS Institute Inc., Cary, NC, USA).

### Data availability

The data that support the findings of this study are available from the Health and Welfare Data Science Center, Ministry of Health and Welfare in Taiwan; however, restrictions apply to the availability of the data, which were used under license for the current study and so are not publicly available. Data are available from the authors upon reasonable request and with permission from the Health and Welfare Data Science Center, Ministry of Health and Welfare in Taiwan.

## Results

A total of 24,101 patients were enrolled in this study, of which 10,609 (44%) were rivaroxaban users and 13,942 were warfarin users. Before the propensity score matching, patients in the rivaroxaban group were older; had higher CHA_2_DS_2_-VASc scores; and had higher rates of VTE, hypertension, and concomitant use of antiplatelet drugs and statins. The demographics and the clinical characteristics of the study groups were all balanced after propensity score matching was performed (Table 1).

**Table 1:** Basic characteristics before and after propensity-score matching^a^.

	Before propensity-score matching			After propensity score-matching		
	Warfarin N = 13,942	Rivaroxaban N = 10,609	Absolute standardized mean difference	Warfarin N = 9,637	Rivaroxaban N = 9,637	Absolute standardized mean difference
Age	70.82 ± 12.73	75.62 ± 10.06	—	74.98 ± 10.60	75.20 ± 10.24	—
<65	4,478 (32.12)	1,351 (12.73)	0.48	1,333 (13.83)	1,351 (14.02)	0.0054
65–69	1,539 (11.04)	1,323 (12.47)	0.04	1,294 (13.43)	1,261 (13.08)	0.0101
70–74	1,833 (13.15)	1,719 (16.20)	0.09	1,586 (16.46)	1,586 (16.46)	0.0031
75–79	2,083 (14.94)	2,115 (19.94)	0.13	1,864 (19.34)	1,855 (19.25)	0.0024
≥80	4,009 (28.75)	4,101 (38.66)	0.21	3,560 (36.94)	3,584 (37.19)	0.0052
Female	5,805 (41.64)	4,849 (45.71)	0.08	4,366 (45.30)	4,378 (45.44)	0.0027
CHA_2_DS_2_-VASc^b^	3.65 ± 2.20	4.06 ± 1.90	—	4.11 ± 2.00	4.02 ± 1.92	—
0	734 (5.26)	116 (1.09)	0.24	119 (1.23)	116 (1.20)	0.0028
1	1,785 (12.80)	670 (6.32)	0.22	629 (6.53)	668 (6.93)	0.0162
≥2	11,423 (81.93)	9,823 (92.59)	0.32	8,889 (92.24)	8,853 (91.86)	0.0138
HAS-BLED^c^	2.23 ± 1.52	2.22 ± 1.45	—	2.33 ± 1.49	2.21 ± 1.46	—
Ischemic stroke/STE	3,236 (23.21)	2,892 (27.26)	0.09	2,599 (26.97)	2,530 (26.25)	0.0162
TIA	671 (4.81)	564 (5.32)	0.02	511 (5.30)	515 (5.34)	0.0018
VTE	567 (4.07)	197 (1.86)	0.13	217 (2.25)	197 (2.04)	0.0143
AMI	670 (4.81)	508 (4.79)	0.001	469 (4.87)	470 (4.88)	0.0005
Heart failure	4,978 (35.71)	3,622 (34.14)	0.03	3,544 (36.77)	3,407 (35.35)	0.0296
Hypertension	9,682 (69.44)	7,891 (74.38)	0.11	7,133 (74.02)	7,106 (73.74)	0.0064
Renal disease	1,770 (12.70)	1,153 (10.87)	0.06	1,154 (11.97)	1,129 (11.72)	0.0080
Liver disease	1,160 (8.32)	763 (7.19)	0.04	705 (7.32)	716 (7.43)	0.0044
DM	4,313 (30.94)	3,282 (30.94)	0	3,064 (31.79)	2,987 (31.00)	0.0172
Peptic ulcer disease	2,635 (18.90)	1,976 (18.63)	0.007	1,848 (19.18)	1,847 (19.17)	0.0003
PVD	777 (5.57)	505 (4.76)	0.04	498 (5.17)	493 (5.12)	0.0023
ICH	255 (1.83)	276 (2.60)	0.05	209 (2.17)	214 (2.22)	0.0035
GI bleeding	999 (7.17)	777 (6.32)	0.006	741 (7.69)	701 (7.27)	0.0158
Coagulation deficiency	24 (0.17)	19 (0.18)	0.002	16 (0.17)	19 (0.20)	0.0073
Antiplatelet drugs	8,639 (61.96)	7,254 (68.38)	0.13	6,418 (66.60)	6,371 (66.11)	0.0103
PPIs	1,447 (10.38)	1,126 (10.61)	0.008	1,017 (10.55)	1,009 (10.47)	0.0027
H2-blockers	3,754 (26.93)	2,807 (26.46)	0.01	2,608 (27.06)	2,587 (26.84)	0.0049
Other antacids	5,601 (40.17)	4,125 (38.88)	0.03	3,924 (40.72)	3,842 (39.87)	0.0173
NSAIDs	6,251 (44.84)	4,547 (42.86)	0.04	4,239 (43.99)	4,207 (43.65)	0.0067
Antiarrhythmic drugs	4,022 (28.85)	3,068 (28.92)	0.002	2,787 (28.92)	2,772 (28.76)	0.0034
Digoxin	2,938 (21.07)	1,872 (17.65)	0.09	1,870 (19.40)	1,806 (18.74)	0.0169
Beta-blockers	6,603 (47.36)	5,054 (47.64)	0.006	4,572 (47.44)	4,529 (47.44)	0.0089
Non-DHP-CCBs	2,885 (20.69)	2,288 (21.57)	0.02	2,075 (21.53)	2,035 (21.12)	0.0101
DHP-CCBs	4,498 (32.26)	3,380 (31.86)	0.009	3,164 (32.83)	3,142 (32.60)	0.0049
ARBs/ACEIs	516 (3.70)	320 (3.02)	0.04	330 (3.42)	311 (3.23)	0.0110
Statins	2,906 (20.84)	2,935 (27.67)	0.16	2,324 (24.12)	2,325 (24.13)	0.0002
Anti-diabetes drugs	3,647 (26.16)	2,777 (26.18)	0.003	2,582 (26.79)	2,526 (26.21)	0.0132

^a^Data was expressed as mean ± standard deviation or number (percentage) unless specified otherwise. The co-morbid diseases were identified from diagnoses within 1 year before the first date of NOAC prescription.

^b^CHA_2_DS_2_-VASc scores ranged from 0 to 9; a higher score indicates a higher risk of stroke or thromboembolism. One point was assigned for congestive heart failure, hypertension, age between 65–74 years, diabetes mellitus, and vascular disease. Two points were assigned for age ≥ 75 years, previous stroke, transient ischemic attack, and systemic thromboembolism.

^c^HAS-BLED scores ranged from 0 to 9; a higher score indicating a higher risk for major bleeding. One point was assigned for hypertension, renal disease, liver disease, stroke, bleeding, age > 65 years, treatment with platelet inhibitors or non-steroidal anti-inflammatory drugs, and alcohol abuse.

Abbreviations: ACEIs: angiotensin converting enzyme inhibitors; AMI: acute myocardial infarctions; ARBs: angiotensin receptor II blockers; CCBs: calcium channel blockers; DHP: dihydropyridine; DM: diabetes mellitus; GI: gastrointestinal; H2: histamine receptor 2; ICH: intracranial hemorrhage; NSAIDs: non-steroidal anti-inflammatory drugs; PPIs: proton pump inhibitors; PVD: peripheral vascular disease; STE: systemic thromboembolism; TIA: transient ischemic attack; VTE: venous thromboembolism.

After propensity score matching, the final sample contained a total of 9,637 rivaroxaban-warfarin matched pairs. The average follow-up duration was 0.96 years for the rivaroxaban group and 1.18 years for the warfarin group. The observed event rates and the hazard ratios (HRs) of the rivaroxaban and warfarin groups after propensity score matching are displayed in Table 2. Overall, rivaroxaban was associated with a significantly lower risk of composite effectiveness outcomes (HR: 0.79; 95% confidence interval [CI]: 0.66–0.94, P = 0.01) and safety outcomes (HR 0.83; 95% CI: 0.71–0.97, P = 0.02) than warfarin. In detail, rivaroxaban was associated with a significantly lower risk of VTE (HR: 0.51; 95% CI: 0.29–0.92, P = 0.02) and ICH (HR: 0.48; 95% CI: 0.32–0.72, P < 0.001) and a comparable risk for ischemic stroke, TIA, GI bleeding, and other bleeding.

**Table 2:** Event numbers, incidence rates, and hazard ratios comparing effectiveness and safety outcomes of warfarin and rivaroxaban use.

	Events (N)		Incidence rate per 100 person-years (95% CI)		HR (95% CI)^a^	P-value
	Warfarin (N = 9,637)	Rivaroxaban (N = 9,637)	Warfarin (N = 9,637)	Rivaroxaban (N = 9,637)		
Composite of effectiveness^b^	317	201	2.79 (2.76–2.83)	2.54 (2.50–2.60)	0.79 (0.66–0.94)	0.01
Ischemic stroke	222	140	1.96 (1.93–1.98)	1.77 (1.74–1.80)	0.80 (0.65–1.00)	0.05
TIA	60	44	0.53 (0.52–0.54)	0.56 (0.54–0.57)	0.90 (0.61–1.34)	0.61
VTE	35	17	0.31 (0.30–0.32)	0.21 (0.20–0.22)	0.51 (0.29–0.92)	0.02
Composite of safety^c^	421	273	3.70 (3.67–3.74)	3.46 (3.42–3.50)	0.83 (0.71–0.97)	0.02
ICH	88	32	0.77 (0.76–0.79)	0.41 (0.39–0.42)	0.48 (0.32–0.72)	<0.001
GI bleeding	186	135	1.63 (1.61–1.66)	1.71 (1.68–1.74)	0.93 (0.74–1.17)	0.54
Other bleeding	147	106	1.29 (1.27–1.31)	1.34 (1.32–1.37)	0.91 (0.71–1.17)	0.47

^a^Hazard ratios were estimated by the Cox proportional hazard model with the warfarin group serving as a reference. Propensity-score matching hazard ratios were controlled by age, sex, annual stroke risk, history of ischemic stroke/systemic embolism, TIA, VTE, acute myocardial infarction, heart failure, hypertension, renal disease, liver disease, diabetes mellitus, peptic ulcer disease, peripheral vascular disease, ICH, GI bleeding, coagulation deficiency, use of antiplatelet, proton pump inhibitors, histamine-2 receptor antagonists, antacids, non-steroid anti-inflammatory drugs, antiarrhythmic drugs, digoxin, beta-blockers, calcium channel blockers, angiotensin converting enzyme inhibitors/angiotensin receptor II blockers, statins, and anti-diabetes drugs.

^b^Composite of effectiveness was the outcome of a composite of ischemic stroke, TIA, and VTE events.

^c^Composite of safety was the outcome of a composite of ICH, GI bleeding, and other bleeding.

Abbreviations: CI: confidence interval; GI: gastrointestinal; HR: hazard ratio; ICH: intracranial hemorrhage; NOAC: non-vitamin K antagonist oral anticoagulants; TIA: transient ischemic attack; VTE: venous thromboembolism.

**Figure 1:**
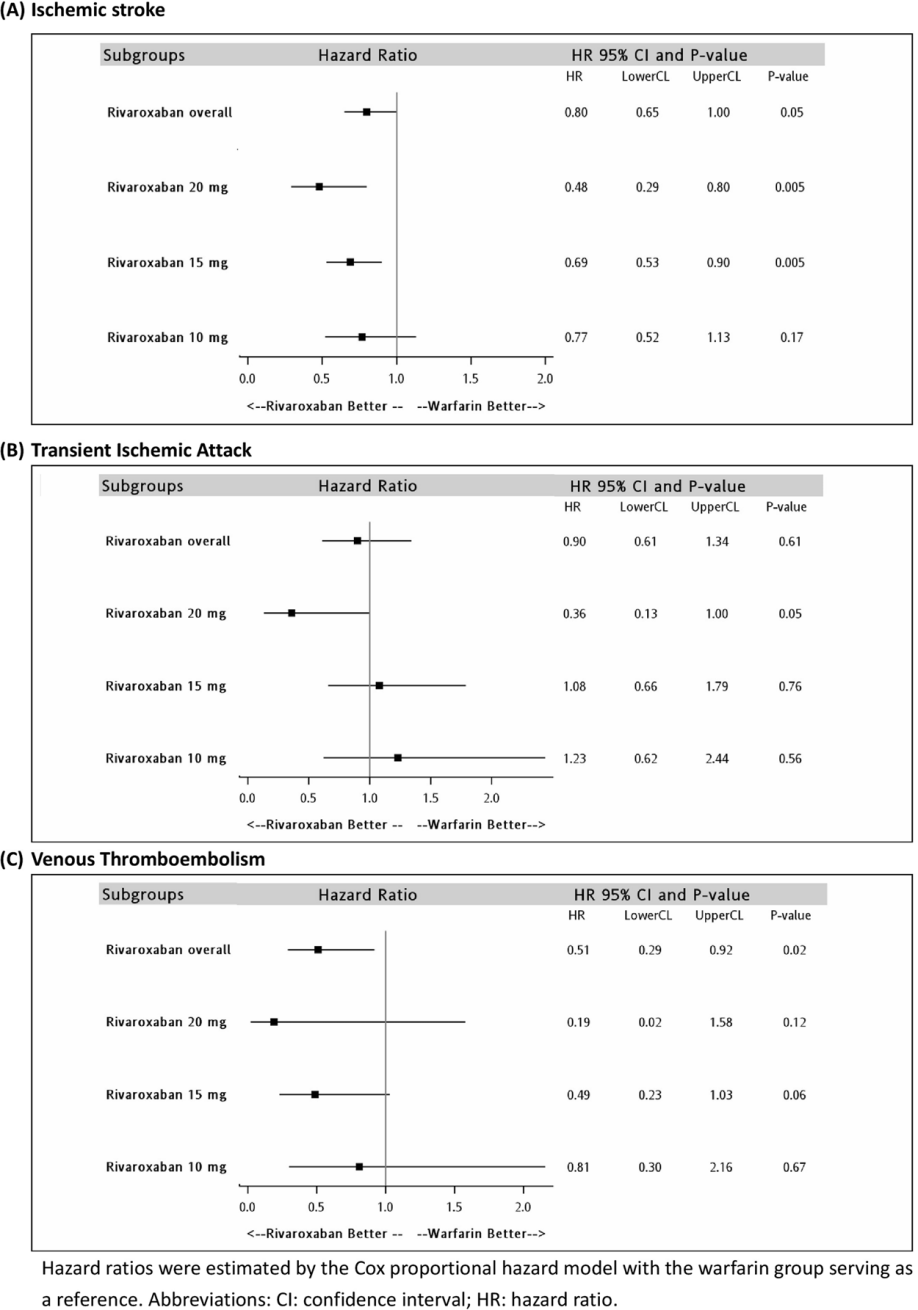
Comparisons of the effectiveness outcomes among different dose regimens in the rivaroxaban group.

**Figure 2:**
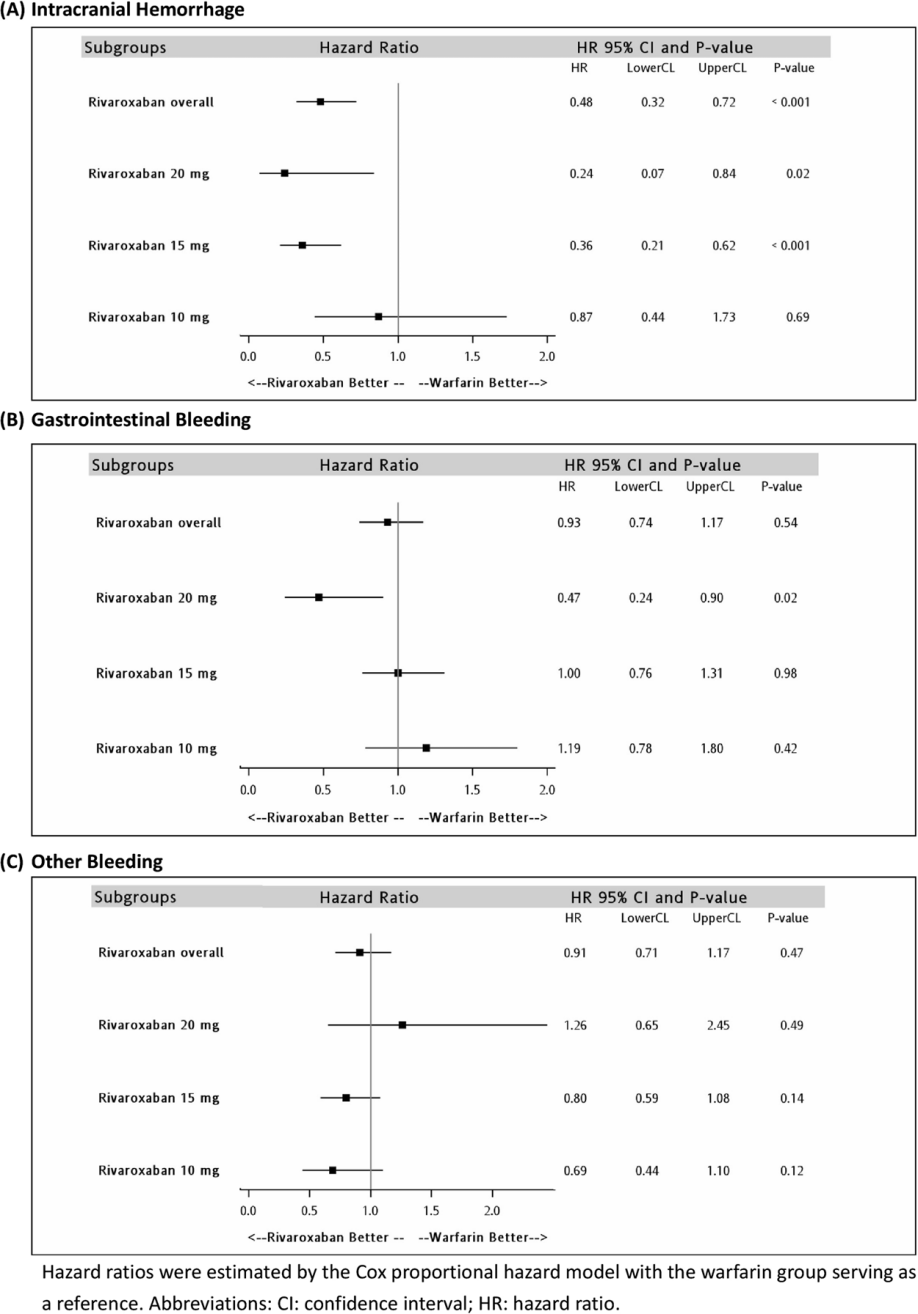
Comparisons of the safety outcomes among different dose regimens in the rivaroxaban group.

When we stratified our analysis by dose, we identified 1,509 pairs in the rivaroxaban 20 mg group, 5,996 pairs in the rivaroxaban 15 mg group, and 3,104 pairs in the rivaroxaban 10 mg group. The baseline characteristics of each group were all balanced after propensity score matching (Supplementary Tables S1 to S4). The adjusted HR among different doses of rivaroxaban and warfarin are displayed in Table 3. Use of rivaroxaban at doses of 20 mg and 15 mg was associated with a significantly lower risk of ischemic stroke (20 mg, HR: 0.48; 95% CI: 0.29–0.80, P = 0.005; 15 mg, HR: 0.69; 95% CI: 0.53–0.90, P = 0.005) and ICH (20 mg, HR: 0.24; 95% CI: 0.07–0.84, P = 0.03; 15 mg, HR: 0.36; 95% CI: 0.21–0.62, P < 0.001). Moreover, treatment with 20 mg rivaroxaban was associated with a lower risk of GI bleeding (HR: 0.47; 95% CI: 0.24–0.90, P = 0.02). In contrast, treatment with 10 mg rivaroxaban did not result in significant differences with regard to any of the effectiveness and safety outcomes. Figures 1 and 2 demonstrate the comparisons of effectiveness and safety outcomes between different doses of rivaroxaban.

**Table 3:** Hazard ratios comparing effectiveness and safety outcomes by different rivaroxaban doses prescribed.

HR^a^ (95%CI); P-value	Rivaroxaban 20 mg (N_matched pair_ = 1,509)	Rivaroxaban 15 mg (N_matched pair_ = 5,996)	Rivaroxaban 10 mg (N_matched pair_ = 3,104)
Ischemic stroke	**0.48 (0.29–0.80)**	**0.69 (0.53–0.90)**	0.77 (0.52–1.13)
	**0.005**	**0.005**	0.17
TIA	0.36 (0.13–1.00)	1.08 (0.66–1.79)	1.23 (0.62–2.44)
	0.05	0.76	0.56
VTE	0.19 (0.02–1.58)	0.49 (0.23–1.03)	0.81 (0.30–2.16)
	0.12	0.06	0.67
ICH	**0.24 (0.07–0.84)**	**0.36 (0.21–0.62)**	0.87 (0.44–1.73)
	**0.02**	**<0.001**	0.69
GI bleeding	**0.47 (0.24–0.90)**	1.00 (0.76–1.31)	1.19 (0.78–1.80)
	**0.02**	0.98	0.42
Other bleeding	1.26 (0.65–2.45)	0.80 (0.59–1.08)	0.69 (0.44–1.10)
	0.49	0.14	0.12

^a^Hazard ratios were estimated by the Cox proportional hazard model with the warfarin group serving as a reference. Propensity-score matching hazard ratios were controlled by age, sex, annual stroke risk, history of ischemic stroke/systemic embolism, TIA, VTE, acute myocardial infarction, heart failure, hypertension, renal disease, liver disease, diabetes mellitus, peptic ulcer disease, peripheral vascular disease, ICH, GI bleeding, coagulation deficiency, use of antiplatelet, proton pump inhibitors, histamine-2 receptor antagonist, antacids, non-steroid anti-inflammatory drugs, antiarrhythmic drugs, digoxin, beta-blockers, calcium channel blockers, angiotensin converting enzyme inhibitors/angiotensin receptor II blockers, statins, and anti-diabetes drugs.

Abbreviations: CI: confidence interval; GI: gastrointestinal; HR: hazard ratio; ICH: intracranial hemorrhage; NOAC: non-vitamin K antagonist oral anticoagulants; TIA: transient ischemic attack; VTE: venous thromboembolism.

**Figure 3:**
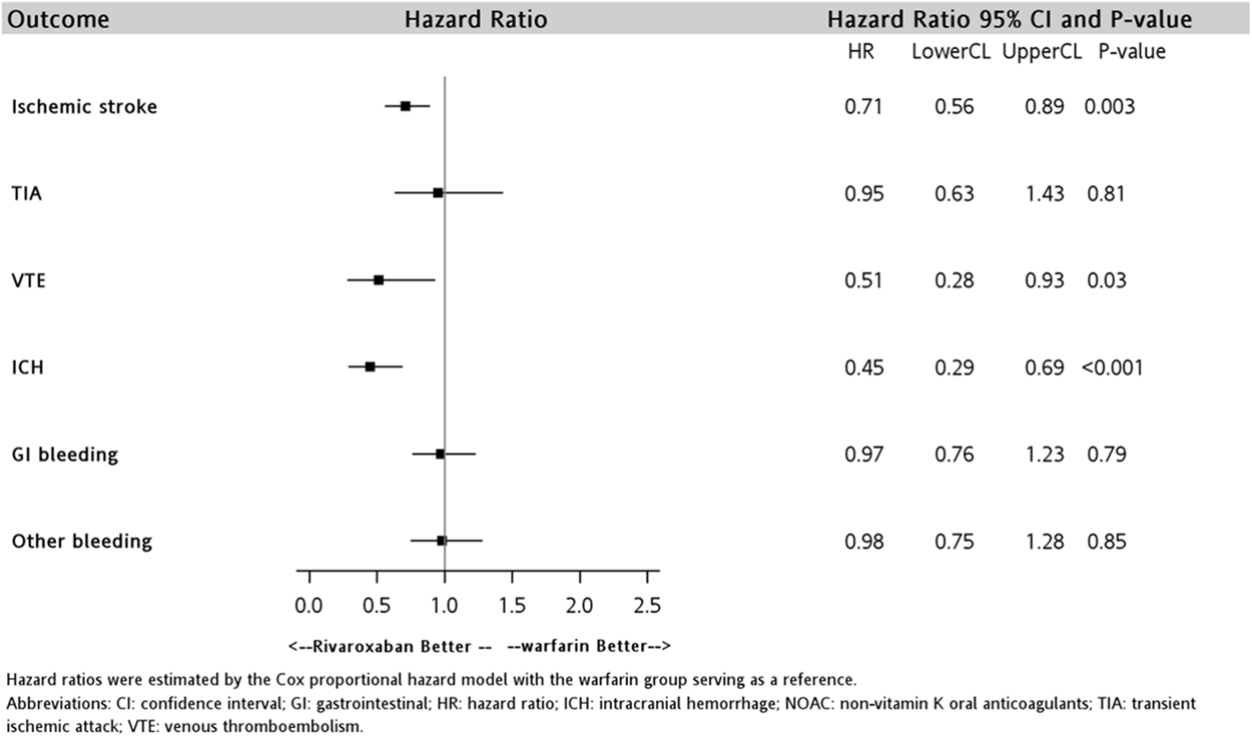
Hazard ratios comparing effectiveness and safety outcomes of rivaroxaban use in patients aged at least 65 years.

**Figure 4:**
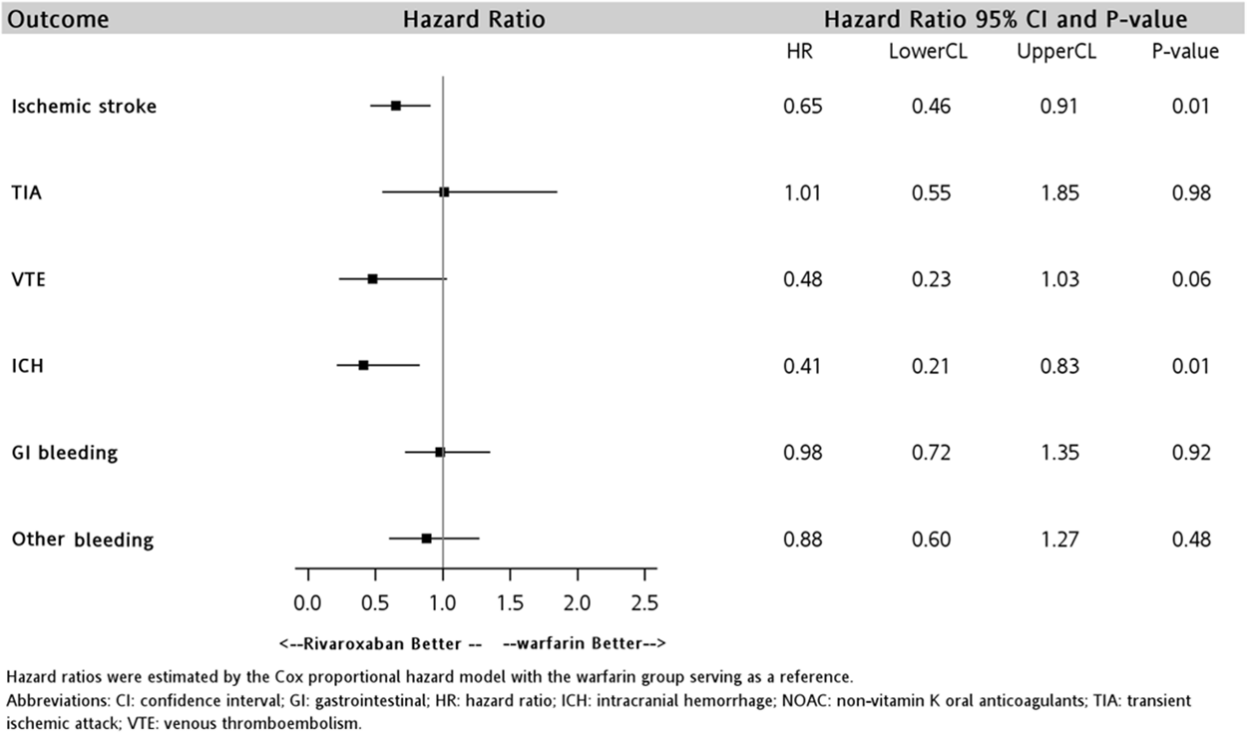
Hazard ratios comparing effectiveness and safety outcomes of rivaroxaban use in patients aged at least 80 years.

The results of subgroup analyses among the elderly patients are displayed in Figs 3 and 4. The risk of ischemic stroke and ICH was significantly lower among rivaroxaban users versus warfarin users in the two age groups. The HR for ischemic stroke was 0.71 (95% CI: 0.56–0.89, P = 0.003) in patients aged ≥65 years and 0.65 (95% CI: 0.46–0.91, P = 0.01) in patients aged ≥80 years. The HR of ICH was 0.45 (95% CI: 0.29–0.69, P = <0.001) and 0.41 (95% CI: 0.21–0.83, P = 0.01) in the group of patients aged ≥65 years and ≥80 years, respectively. Notably, rivaroxaban was associated with a significantly lower risk of VTE (HR: 0.51; 95% CI: 0.28–0.93, P = 0.03) only among users aged ≥65 years.

## Discussion

To the best of our knowledge, this is the first population-based observational study, which focuses specifically on Asians to assess the effectiveness and safety of rivaroxaban at different dosage regimens. In the present study, we observed a significantly lower risk of VTE and ICH for rivaroxaban compared to warfarin. Furthermore, rivaroxaban at a dose ≥15 mg was associated with a significantly lower risk of ischemic stroke. The risk of GI bleeding was generally comparable between rivaroxaban and warfarin users across all different dosage and age groups, except for the rivaroxaban 20 mg group, which showed a significantly lower risk of GI bleeding than the warfarin group.

Before propensity score matching, patients in the rivaroxaban group were older, more commonly diagnosed with systemic thromboembolism and hypertension, and had higher CHA_2_DS_2_-VASc scores than the warfarin group. This can be attributed to the reimbursement criteria for the NOACs in the NHI program: patients need to be ≥75 years old, have a history of ischemic stroke, systemic thromboembolism, or congestive heart failure. For patients aged between 65 and 74 years, at least one diagnosis of diabetes, hypertension, or coronary artery disease is required. The majority of patients in this study were prescribed low dose rivaroxaban (15 mg and 10 mg). The reasons for this prescription pattern are multifocal. First, Asians, compared to non-Asians, are more liable to experience warfarin-related bleeding, even under lower intensity anticoagulant therapy^27^. Therefore, physicians tend to be more conservative in Taiwan. Second, patients under NOAC therapy in Taiwan were older than warfarin users due to the restrictions in the NHI program. Elderly patients were more prone to receive a renal adjusted dose, as the glomerular filtration rate reduces with age. Third, physicians may prescribe rivaroxaban according to the Japanese labelling^6^.

Regarding the effectiveness, we observed a dose-related response. As the rivaroxaban dose increased from 10 to 15 and 20 mg, the risk of ischemic stroke became significantly lower than for warfarin and the risk reduction was even more prominent for the 20 mg group. This suggests that in the Asian population, the standard dosing regimen (i.e., 20 mg daily) is potentially more appropriate and should be considered in patients with no concern of increased bleeding risk. In the ROCKET AF and J-ROCKET AF study, however, the risk of ischemic stroke was comparable between the rivaroxaban and warfarin groups. Patients in the present study had less baseline risk for thromboembolism as indicated by lower average CHADS2 scores (the present study, 2.64; J-ROCKET AF, 3.27; ROCKET AF, 3.2), which may explain the more prominent effectiveness of rivaroxaban in our data. We also observed a significantly lower risk of VTE in the rivaroxaban group. AF has been shown to increase the risk of future development of VTE^28^. The efficacy of rivaroxaban in secondary VTE prevention has been proven previously in a well-controlled study^29^. Our results imply that rivaroxaban treatment is effective in preventing VTE among AF patients; however, further prospective studies that exclusively enrol AF patients may be warranted.

Regarding safety as reported in the ROCKET AF study, we observed a significantly lower risk of ICH and a comparable risk of GI bleeding in patients treated with rivaroxaban. In addition, our data showed that 20 mg was paradoxically associated with a reduced risk of GI bleeding. In contrast, rivaroxaban 10 mg did not significantly reduce the risk of ICH. These results may be explained by the between-group variation. In the present study, physicians determined the dosage regimen based on their judgment in the clinical setting. Therefore, patients in the rivaroxaban 20 mg group tended to be more robust, have a lower risk of bleeding as indicated by lower HAS-BLED scores, and experienced fewer GI bleeding episodes. On the other hand, patients in the rivaroxaban 10 mg group were more fragile, as indicated by older age, a higher prevalence of renal dysfunction, hypertension, and higher CHA_2_DS_2_-VASc and HAS-BLED scores, which led to a less prominent reduction in the risk for ICH.

The results of the subgroup analysis for the elderly patients were consistent with the main analysis. Rivaroxaban was associated with a lower risk of VTE and ICH than warfarin. The prominent risk reduction associated with rivaroxaban in ischemic stroke did not change in the elderly population. Elderly patients generally have a higher risk of ischemic stroke, systemic thromboembolism, and bleeding, and a higher prevalence of comorbidities and polypharmacy, but lower mobility for frequent laboratory monitoring^30,31^. Our results also indicated that even in patients of advanced age (i.e., age ≥80), rivaroxaban use remains effective and safe. Rivaroxaban may be an alternative to warfarin given its superior effectiveness profile and lower risk of ICH for both elderly (i.e., age ≥65) and very elderly (i.e., age ≥80) patients.

We acknowledge the following limitations in our study. First, laboratory results were not available in the claims data from the NHI program; therefore, we were unable to evaluate the proportion of time spent in the therapeutic international normalized ratio (INR) range (i.e., time in therapeutic range, TTR). Sub-optimal TTR in the warfarin group can lead to an over-estimation of the effectiveness of rivaroxaban. However, we censored the patients who discontinued oral anticoagulation therapy or had a > 30-day gap between the end of an oral anticoagulant prescription and the next prescription to ensure persistent use of oral anticoagulants. We assumed that the persistent use of warfarin would reflect a minimum level of anticoagulant use and therefore ensured a certain level of time in therapeutic range in warfarin users. Second, we did not directly compare the outcomes across different rivaroxaban dose groups. The dose relationship was a result of an indirect comparison and thus can potentially suffer from selection bias due to the different baseline characteristics of the three dose groups. Given that the paradoxical relationship between rivaroxaban dose and the risk of ICH, we suspected that unmeasured variables may possibly exist. Therefore, some biases may still remain in the results in spite of propensity score matching. Third, we were unable to evaluate the adherence. Nevertheless, patients who did not refill their medications for more than 30 days were censored from this study. With an uncertain status of adherence, our data still showed improved effectiveness and an improved safety profile with rivaroxaban. Finally, only baseline demographics within 12 months before the index date were included for propensity score matching. Medical conditions that occurred more than one year prior to the index date were not taken into consideration, which may have resulted in an underestimation of the baseline risk for thrombosis and bleeding. Nevertheless, previous studies have applied the 12 month period to validate the CHA_2_DS_2_-VASc scoring system^32^, and the same approach has also been used in several observational studies investigating the effectiveness and safety of NOACs^10,23,33^. Comorbidities, such as heart failure, hypertension, and diabetes mellitus, were considered as chronic diseases and would be detected by claims data as long as patients re-visited their physician and refilled medications. Since patients with chronic conditions generally need long-term management, it is most likely that these patients will continuously use health services and thus be recorded in the claims database. Therefore, we should be able to identify the pre-existing conditions correctly even if the conditions were first diagnosed outside the one-year per-index period.

In conclusion, rivaroxaban was associated with a lower risk of VTE and ICH than warfarin in patients with non-valvular AF. At doses over 15 mg, rivaroxaban was associated with a significantly lower risk of ischemic stroke. Our study supports rivaroxaban as an effective and safe choice even among the elderly population.

## Electronic supplementary material

Supplementart Tables S1 to S4
