# Supplementary material for: Effectiveness and Safety of Different Rivaroxaban Dosage Regimens in Patients with Non-Valvular Atrial Fibrillation: A Nationwide, Population-Based Cohort Study
Source: Sci Rep. 2018 Feb 22;8:3451. doi: 10.1038/s41598-018-21884-y (PMC5823875; doi:10.1038/s41598-018-21884-y)
Supplement: Supplementary file 1 — Supplementart Tables S1 to S4 [file 41598_2018_21884_MOESM1_ESM.docx]

**Effectiveness and Safety of Different Rivaroxaban Dosage Regimens
in Patients with Non-valvular Atrial Fibrillation:
A Nationwide, Population-Based Cohort Study**Hsin-Yi Huang, M.S.^1,2^, Shin-Yi Lin, M.S.^3,4^, Shou-Hsia Cheng, Ph.D.^5^,

Chi-Chuan Wang, Ph.D.^3,4,*^

1. Graduate Institute of Clinical Pharmacy, College of Pharmacy, National Taiwan University, Taipei, Taiwan.
2. Department of Pharmacy, Shuang Ho Hospital, Taipei Medical University
3. School of Pharmacy, National Taiwan University, Taipei, Taiwan
4. Department of Pharmacy, National Taiwan University Hospital, Taipei, Taiwan
5. Institute of Health Policy and Management, College of Public Health, National Taiwan University, Taipei, Taiwan

Corresponding Author:

*Chi-Chuan Wang, Ph.D.

School of Pharmacy, National Taiwan University

Address: 2F, No.33, Linsen S. Rd., Zhongsheng Dist., Room 203

Taipei 10050, Taiwan

Phone: 886-2-3366-8739

Fax: 886-2-3366-8739

Email: [chicwang@ntu.edu.tw](mailto:chicwang@ntu.edu.tw)

| **Supplementary Table S1. Basic characteristics of rivaroxaban 20mg users after propensity-score matching^a^** | | | |
| --- | --- | --- | --- |
|  | **Warfarin N=1,509** | **Rivaroxaban 20mg  N=1,509** | **Absolute standardized**  **mean difference** |
| **Age** | 73.79 ± 10.85 | 73.72 ± 10.71 | - |
| **< 65** | 249 (16.50) | 261 (17.30) | 0.0212 |
| **65-69** | 236 (15.64) | 233 (15.44) | 0.0055 |
| **70-74** | 272 (18.03) | 262 (17.36) | 0.0013 |
| **75-79** | 263 (17.43) | 254 (16.83) | 0.0158 |
| **≥ 80** | 489 (32.41) | 499 (33.07) | 0.0141 |
| **Female** | 604 (40.03) | 620 (41.09) | 0.0216 |
| **CHA_2_DS_2_-VASc**^b^ | 3.81 ± 1.94 | 3.79 ± 1.84 | - |
| **0** | 25 (1.66) | 24 (1.59) | 0.0052 |
| **1** | 119 (7.89) | 117 (7.75) | 0.0049 |
| **≥2** | 1,365 (90.46) | 1,368 (90.66) | 0.0068 |
| **HAS-BLED**^c^ | 2.17 ± 1.45 | 2.12 ± 1.41 | - |
| **Ischemic stroke/STE** | 358 (23.72) | 366 (24.25) | 0.0124 |
| **TIA** | 57 (3.78) | 64 (4.24) | 0.0236 |
| **VTE** | 28 (1.86) | 25 (1.66) | 0.0151 |
| **AMI** | 74 (4.90) | 76 (5.04) | 0.0061 |
| **Heart failure** | 530 (35.12) | 538 (35.65) | 0.0111 |
| **Hypertension** | 1,091 (72.30) | 1,083 (71.77) | 0.0118 |
| **Renal disease** | 142 (9.41) | 154 (10.21) | 0.0267 |
| **Liver disease** | 93 (6.16) | 106 (7.02) | 0.0347 |
| **DM** | 479 (31.74) | 474 (31.41) | 0.0071 |
| **Peptic ulcer disease** | 251 (16.63) | 257 (17.03) | 0.0106 |
| **PVD** | 62 (4.11) | 59 (3.91) | 0.0101 |
| **ICH** | 37 (2.45) | 42 (2.78) | 0.0208 |
| **GI bleeding** | 86 (5.70) | 97 (6.43) | 0.0305 |

| **Supplementary Table S1 (Continued).**  **Basic characteristics of rivaroxaban 20mg users after propensity-score matching** | | | |
| --- | --- | --- | --- |
|  | **Warfarin N=1,509** | **Rivaroxaban 20mg  N=1,509** | **Absolute standardized**  **mean difference** |
| **Coagulation deficiency** | 2 (0.13) | 5 (0.33) | 0.0413 |
| **Antiplatelet drugs** | 1,026 (67.99) | 1,030 (68.26) | 0.0109 |
| **PPIs** | 153 (10.14) | 158 (10.47) | 0.0057 |
| **H2-blockers** | 333 (22.07) | 361 (23.92) | 0.0441 |
| **Other antacids** | 606 (40.16) | 590 (39.10) | 0.0217 |
| **NSAIDs** | 675 (44.73) | 677 (44.86) | 0.0027 |
| **Antiarrhythmic drugs** | 417 (27.63) | 425 (28.16) | 0.0118 |
| **Digoxin** | 272 (18.03) | 271 (17.96) | 0.0017 |
| **Beta-blockers** | 711 (47.12) | 727 (48.18) | 0.0212 |
| **Non-DHP-CCBs** | 334 (22.13) | 356 (23.59) | 0.0347 |
| **DHP-CCBs** | 455 (30.15) | 445 (29.49) | 0.0145 |
| **ARBs/ACEIs** | 29 (1.92) | 38 (2.52) | 0.0405 |
| **Statins** | 409 (27.10) | 436 (28.89) | 0.0399 |
| **Anti-diabetes drugs** | 400 (26.51) | 402 (26.64) | 0.0030 |
| ^a^ Data was expressed as mean ± standard deviation or number (percentage) unless specified otherwise. The co-morbid diseases were identified from diagnoses within 1 year before the first date of NOAC prescription.  ^b^ CHA_2_DS_2_-VASc scores ranged from 0 to 9; a higher score indicates a higher risk of stroke or thromboembolism. One point was assigned for congestive heart failure, hypertension, age between 65-74 years, diabetes mellitus, and vascular disease. Two points were assigned for age ≥ 75 years, previous stroke, transient ischemic attack, and systemic thromboembolism.  ^c^ HAS-BLED scores ranged from 0 to 9; a higher score indicating a higher risk for major bleeding. One point was assigned for hypertension, renal disease, liver disease, stroke, bleeding, age > 65 years, treatment with platelet inhibitors or non-steroidal anti-inflammatory drugs, and alcohol abuse.  **Abbreviations:** ACEIs: angiotensin converting enzyme inhibitors; AMI: acute myocardial infarctions; ARBs: angiotensin receptor II blockers; CCBs: calcium channel blockers; DHP: dihydropyridine; DM: diabetes mellitus; GI: gastrointestinal; H2: histamine receptor 2; ICH: intracranial hemorrhage; NSAIDs: non-steroidal anti-inflammatory drugs; PPIs: proton pump inhibitors; PVD: peripheral vascular disease; STE: systemic thromboembolism; TIA: transient ischemic attack; VTE: venous thromboembolism. | | | |

| **Supplementary Table S2 . Basic characteristics of rivaroxaban 15mg users after propensity-score matching^a^** | | | |
| --- | --- | --- | --- |
|  | **Warfarin N=5,996** | **Rivaroxaban 15mg N=5,996** | **Absolute standardized**  **mean difference** |
| **Age** | 75.03 ± 10.39 | 74.93 ± 10.00 | - |
| **< 65** | 818 (13.64) | 845 (14.09) | 0.0130 |
| **65-69** | 755 (12.59) | 785 (13.09) | 0.0150 |
| **70-74** | 1,016 (16.94) | 1,005 (16.76) | 0.0155 |
| **75-79** | 1,220 (20.35) | 1,252 (20.88) | 0.0132 |
| **≥ 80** | 2,187 (36.47) | 2,109 (35.17) | 0.0271 |
| **Female** | 2,709 (45.18) | 2,687 (44.81) | 0.0074 |
| **CHA_2_DS_2_-VASc**^b^ | 4.22 ± 2.00 | 4.04 ± 1.93 | - |
| **0** | 68 (1.13) | 67 (1.12) | 0.0016 |
| **1** | 401 (6.69) | 422 (7.04) | 0.0139 |
| **≥2** | 5,527 (92.18) | 5,507 (91.84) | 0.0123 |
| **HAS-BLED**^c^ | 2.32 ± 1.51 | 2.24±1.45 | - |
| **Ischemic stroke/STE** | 1,736 (28.95) | 1,706 (28.45) | 0.0111 |
| **TIA** | 316 (5.27) | 319 (5.32) | 0.0022 |
| **VTE** | 142 (2.37) | 136 (2.27) | 0.0066 |
| **AMI** | 265 (4.42) | 258 (4.30) | 0.0057 |
| **Heart failure** | 2,010 (33.52) | 1,978 (32.99) | 0.0113 |
| **Hypertension** | 4,546 (75.82) | 4,480 (74.72) | 0.0255 |
| **Renal disease** | 634 (10.57) | 624 (10.41) | 0.0054 |
| **Liver disease** | 429 (7.15) | 442 (7.37) | 0.0084 |
| **DM** | 1,889 (31.50) | 1,883 (31.40) | 0.0022 |
| **Peptic ulcer disease** | 1,084 (18.08) | 1,114 (18.58) | 0.0129 |
| **PVD** | 290 (4.84) | 279 (4.65) | 0.0086 |
| **ICH** | 142 (2.37) | 156 (2.60) | 0.0150 |
| **GI bleeding** | 417 (6.95) | 432 (7.20) | 0.0098 |

| **Supplementary Table S2 (Continued).**  **Basic characteristics of rivaroxaban 15mg users after propensity-score matching** | | | |
| --- | --- | --- | --- |
|  | **Warfarin N=5,996** | **Rivaroxaban 15mg N=5,996** | **Absolute standardized**  **mean difference** |
| **Coagulation deficiency** | 8 (0.13) | 8 (0.13) | 0.0000 |
| **Antiplatelet drugs** | 4,097 (68.33) | 4,134 (68.95) | 0.0133 |
| **PPIs** | 625 (10.42) | 644 (10.74) | 0.0103 |
| **H2-blockers** | 1,588 (26.48) | 1,575 (26.27) | 0.0049 |
| **Other antacids** | 2,324 (38.76) | 2,321 (38.71) | 0.0010 |
| **NSAIDs** | 2,557 (42.65) | 2,597 (43.31) | 0.0135 |
| **Antiarrhythmic drugs** | 1,675 (27.94) | 1,695 (28.27) | 0.0074 |
| **Digoxin** | 1,094 (18.25) | 1,082 (18.05) | 0.0052 |
| **Beta-blockers** | 2,825 (47.11) | 2,860 (47.70) | 0.0117 |
| **Non-DHP-CCBs** | 1,270 (21.18) | 1,281 (21.36) | 0.0045 |
| **DHP-CCBs** | 1,917 (31.97) | 1,897 (31.64) | 0.0072 |
| **ARBs/ACEIs** | 198 (3.30) | 181 (3.02) | 0.0162 |
| **Statins** | 1,618 (26.98) | 1,642 (27.38) | 0.0090 |
| **Anti-diabetes drugs** | 1,582 (26.38) | 1,595 (26.60) | 0.0049 |
| ^a^ Data was expressed as mean ± standard deviation or number (percentage) unless specified otherwise. The co-morbid diseases were identified from diagnoses within 1 year before the first date of NOAC prescription.  ^b^ CHA_2_DS_2_-VASc scores ranged from 0 to 9; a higher score indicates a higher risk of stroke or thromboembolism. One point was assigned for congestive heart failure, hypertension, age between 65-74 years, diabetes mellitus, and vascular disease. Two points were assigned for age ≥ 75 years, previous stroke, transient ischemic attack, and systemic thromboembolism.  ^c^ HAS-BLED scores ranged from 0 to 9; a higher score indicating a higher risk for major bleeding. One point was assigned for hypertension, renal disease, liver disease, stroke, bleeding, age > 65 years, treatment with platelet inhibitors or non-steroidal anti-inflammatory drugs, and alcohol abuse.  **Abbreviations:** ACEIs: angiotensin converting enzyme inhibitors; AMI: acute myocardial infarctions; ARBs: angiotensin receptor II blockers; CCBs: calcium channel blockers; DHP: dihydropyridine; DM: diabetes mellitus; GI: gastrointestinal; H2: histamine receptor 2; ICH: intracranial hemorrhage; NSAIDs: non-steroidal anti-inflammatory drugs; PPIs: proton pump inhibitors; PVD: peripheral vascular disease; STE: systemic thromboembolism; TIA: transient ischemic attack; VTE: venous thromboembolism. | | | |

| **Supplementary Table S3 . Basic characteristics of rivaroxaban 10mg users after propensity-score matching^a^** | | | |
| --- | --- | --- | --- |
|  | **Warfarin N=3,104** | **Rivaroxaban 10mg   N=3,104** | **Absolute standardized**  **mean difference** |
| **Age** | 77.69 ± 9.33 | 77.88 ± 9.45 | - |
| **< 65** | 243 (7.83) | 245 (7.89) | 0.0024 |
| **65-69** | 325 (10.47) | 305 (9.83) | 0.0213 |
| **70-74** | 430 (13.85) | 452 (14.56) | 0.0028 |
| **75-79** | 606 (19.52) | 609 (19.62) | 0.0024 |
| **≥ 80** | 1,500 (48.32) | 1,493 (48.10) | 0.0045 |
| **Female** | 1,549 (49.90) | 1,542 (49.68) | 0.0045 |
| **CHA_2_DS_2_-VASc**^b^ | 4.25 ± 1.89 | 4.24 ± 1.88 | - |
| **0** | 20 (0.64) | 25 (0.81) | 0.0190 |
| **1** | 126 (4.06) | 131 (4.22) | 0.0081 |
| **≥2** | 2,958 (95.30) | 2,948 (94.97) | 0.0150 |
| **HAS-BLED**^c^ | 2.29 ± 1.49 | 2.24 ± 1.44 | - |
| **Ischemic stroke/STE** | 817 (26.32) | 820 (26.42) | 0.0022 |
| **TIA** | 171 (5.51) | 181 (5.83) | 0.0139 |
| **VTE** | 37 (1.19) | 36 (1.16) | 0.0030 |
| **AMI** | 159 (5.12) | 174 (5.61) | 0.0214 |
| **Heart failure** | 1,067 (34.38) | 1,106 (35.63) | 0.0263 |
| **Hypertension** | 2,323 (74.84) | 2,328 (75.00) | 0.0037 |
| **Renal disease** | 376 (12.11) | 375 (12.08) | 0.0010 |
| **Liver disease** | 218 (7.02) | 215 (6.93) | 0.0038 |
| **DM** | 933 (30.06) | 925 (29.80) | 0.0056 |
| **Peptic ulcer disease** | 616 (19.85) | 605 (19.49) | 0.0089 |
| **PVD** | 157 (5.06) | 167 (5.38) | 0.0145 |
| **ICH** | 79 (2.55) | 78 (2.51) | 0.0021 |
| **GI bleeding** | 220 (7.09) | 248 (7.99) | 0.0342 |

| **Supplementary Table S3 (Continued).**  **Basic characteristics of rivaroxaban 10mg users after propensity-score matching** | | | |
| --- | --- | --- | --- |
|  | **Warfarin N=3,104** | **Rivaroxaban 10mg   N=3,104** | **Absolute standardized**  **mean difference** |
| **Coagulation deficiency** | 11 (0.35) | 6 (0.19) | 0.0308 |
| **Antiplatelet drugs** | 2,067 (66.59) | 2,090 (67.33) | 0.0158 |
| **PPIs** | 320 (10.31) | 324 (10.44) | 0.0042 |
| **H2-blockers** | 856 (27.58) | 871 (28.06) | 0.0108 |
| **Other antacids** | 1,218 (39.24) | 1,214 (39.11) | 0.0026 |
| **NSAIDs** | 1,294 (41.69) | 1,273 (41.01) | 0.0137 |
| **Antiarrhythmic drugs** | 959 (30.90) | 948 (30.54) | 0.0077 |
| **Digoxin** | 504 (16.24) | 519 (16.72) | 0.0130 |
| **Beta-blockers** | 1,438 (46.33) | 1,467 (47.26) | 0.0187 |
| **Non-DHP-CCBs** | 603 (19.43) | 651 (20.97) | 0.0385 |
| **DHP-CCBs** | 1,047 (33.73) | 1,038 (33.44) | 0.0061 |
| **ARBs/ACEIs** | 89 (2.87) | 101 (3.25) | 0.0224 |
| **Statins** | 832 (26.80) | 857 (27.61) | 0.0181 |
| **Anti-diabetes drugs** | 795 (25.61) | 780 (25.13) | 0.0111 |
| ^a^ Data was expressed as mean ± standard deviation or number (percentage) unless specified otherwise. The co-morbid diseases were identified from diagnoses within 1 year before the first date of NOAC prescription.  ^b^ CHA_2_DS_2_-VASc scores ranged from 0 to 9; a higher score indicates a higher risk of stroke or thromboembolism. One point was assigned for congestive heart failure, hypertension, age between 65-74 years, diabetes mellitus, and vascular disease. Two points were assigned for age ≥ 75 years, previous stroke, transient ischemic attack, and systemic thromboembolism.  ^c^ HAS-BLED scores ranged from 0 to 9; a higher score indicating a higher risk for major bleeding. One point was assigned for hypertension, renal disease, liver disease, stroke, bleeding, age > 65 years, treatment with platelet inhibitors or non-steroidal anti-inflammatory drugs, and alcohol abuse.  **Abbreviations:** ACEIs: angiotensin converting enzyme inhibitors; AMI: acute myocardial infarctions; ARBs: angiotensin receptor II blockers; CCBs: calcium channel blockers; DHP: dihydropyridine; DM: diabetes mellitus; GI: gastrointestinal; H2: histamine receptor 2; ICH: intracranial hemorrhage; NSAIDs: non-steroidal anti-inflammatory drugs; PPIs: proton pump inhibitors; PVD: peripheral vascular disease; STE: systemic thromboembolism; TIA: transient ischemic attack; VTE: venous thromboembolism. | | | |

| Supplementary Table S4. Basic characteristics of at least 65 and 80 years old elderly users after propensity-score matching^a^ | | | | | | | | | | | | | |
| --- | --- | --- | --- | --- | --- | --- | --- | --- | --- | --- | --- | --- | --- |
|  | | **Aged at least 65 years old** | | | | | | **Aged at least 80 years old** | | | | | |
|  | | **Warfarin N=8,253** | | **Rivaroxaban   N=8,253** | | **Absolute standardized**  **mean difference** | | **Warfarin N=3,458** | | **Rivaroxaban**  **15mg N=3,458** | | **Absolute standardized**  **mean difference** | |
| **Age** | | 77.96 ± 7.50 | | 78.06 ± 7.51 | | - | | 85.18 ± 3.96 | | 85.26 ± 4.00 | | - | |
| **65-69** | | 1,295 (15.69) | | 1,268 (15.36) | | 0.0090 | | - | | - | | - | |
| **70-74** | | 1,583 (19.18) | | 1,588 (19.24) | | 0.0090 | | - | | - | | - | |
| **75-79** | | 1,844 (22.34) | | 1,841 (22.31) | | 0.0009 | | - | | - | | - | |
| **≥80** | | 3,531 (42.78) | | 3,556 (43.09) | | 0.0061 | | - | | - | | - | |
| **Female** | | 3,991 (48.36) | | 3,994 (48.39) | | 0.0007 | | 1,854(53.61) | | 1,842 (53.27) | | 0.0074 | |
| **CHA_2_DS_2_-VASc**^b^ | | 4.36 ±1.89 | | 4.32 ± 1.85 | | - | | 5.01 ± 1.76 | | 4.95 ± 1.75 | | - | |
| **0** | | - | | - (-) | | - | | - | | - | | - | |
| **1** | | 327 (3.96) | | 325 (3.94) | | 0.0012 | | - | | - | | - | |
| **≥2** | | 7,926 (96.04) | | 7,928 (96.06) | | 0.0012 | | - | | - | | - | |
| **HAS-BLED**^c^ | | 2.34 ± 1.51 | | 2.24 ± 1.47 | | - | | 2.58 ±1.49 | | 2.46±1.46 | | - | |
| **Ischemic stroke/STE** | | 2,227 (26.98) | | 2,228 (27.00) | | 0.0003 | | 1,096(31.69) | | 1,067 (30.86) | | 0.0181 | |
| **TIA** | | 468 (5.67) | | 468 (5.67) | | 0.0000 | | 227 (6.56) | | 210 (6.07) | | 0.0202 | |
| **VTE** | | 181 (2.19) | | 176 (2.13) | | 0.0042 | | 111 (3.21) | | 99 (2.86) | | 0.0202 | |
| **AMI** | | 375 (4.54) | | 382 (4.63) | | 0.0041 | | 175 (5.06) | | 170 (4.92) | | 0.0066 | |
| **Heart failure** | | 2990 (36.23) | | 2921 (35.39) | | 0.0174 | | 1,512(43.72) | | 1,503 (43.46) | | 0.0052 | |
| **Hypertension** | | 6210 (75.25) | | 6203 (75.16) | | 0.0020 | | 2,712(78.43) | | 2,697 (77.99) | | 0.0105 | |
| **Renal disease** | | 1071 (12.98) | | 1044 (12.65) | | 0.0098 | | 511 (14.78) | | 506 (14.63) | | 0.0041 | |
| **Liver disease** | | 580 (7.03) | | 571 (6.92) | | 0.0043 | | 189 (5.47) | | 183 (5.29) | | 0.0077 | |
| **DM** | | 2570 (31.14) | | 2584 (31.31) | | 0.0037 | | 1,029(29.76) | | 1,025 (29.64) | | 0.0025 | |
| **Peptic ulcer disease** | | 1665 (20.17) | | 1639 (19.86) | | 0.0079 | | 732 (21.17) | | 719 (20.79) | | 0.0092 | |
| **PVD** | | 469 (5.68) | | 451 (5.46) | | 0.0095 | | 225 (6.51) | | 227 (6.56) | | 0.0023 | |
| **ICH** | | 178 (2.16) | | 171 (2.07) | | 0.0059 | | 69 (2.00) | | 62 (1.79) | | 0.0149 | |
| **GI bleeding** | | 618 (7.49) | | 625 (7.57) | | 0.0032 | | 312 (9.02) | | 307 (8.88) | | 0.0051 | |
| **Coagulation deficiency** | | 17 (0.21) | | 15 (0.18) | | 0.0055 | | 8 (0.23) | | 9 (0.26) | | 0.0058 | |
| **Supplementary Table S4 (Continued).**  **Basic characteristics of at least 65 and 80 years old elderly users after propensity-score matching** | | | | | | | | | | | | |  |
|  | **Aged at least 65 years old** | | | | | | **Aged at least 80 years old** | | | | | |  |
|  | **Warfarin N=8,253** | | **Rivaroxaban   N=8,253** | | **Absolute standardized**  **mean difference** | | **Warfarin N=3,458** | | **Rivaroxaban**  **15mg N=3,458** | | **Absolute standardized**  **mean difference** | |  |
| **Antiplatelet drugs** | 5,396 (65.38) | | 5,418 (65.65) | | 0.0056 | | 2,343(67.76) | | 2,316 (66.98) | | 0.0167 | |  |
| **PPIs** | 894 (10.83) | | 888 (10.76) | | 0.0023 | | 441 (12.75) | | 436 (12.61) | | 0.0043 | |  |
| **H2-blockers** | 2,316 (28.06) | | 2,293 (27.78) | | 0.0062 | | 1,016(29.38) | | 994 (28.74) | | 0.0140 | |  |
| **Other antacids** | 3,461 (41.94) | | 3,384 (41.00) | | 0.0189 | | 1,522(44.01) | | 1,526 (44.13) | | 0.0023 | |  |
| **NSAIDs** | 3,673 (44.51) | | 3,640 (44.11) | | 0.0080 | | 1,465(42.37) | | 1,455 (42.08) | | 0.0059 | |  |
| **Antiarrhythmic drugs** | 2,323 (28.15) | | 2,322 (28.14) | | 0.0003 | | 946 (27.36) | | 936 (27.07) | | 0.0065 | |  |
| **Digoxin** | 1,547 (18.74) | | 1,539 (18.65) | | 0.0025 | | 737 (21.31) | | 734 (21.23) | | 0.0021 | |  |
| **Beta-blockers** | 3,751 (45.45) | | 3,735 (45.26) | | 0.0039 | | 1,514(43.78) | | 1,459 (42.19) | | 0.0321 | |  |
| **Non-DHP-CCBs** | 1750 (21.20) | | 1739 (21.07) | | 0.0033 | | 808 (23.37) | | 809 (23.40) | | 0.0007 | |  |
| **DHP-CCBs** | 2844 (34.46) | | 2818 (34.15) | | 0.0066 | | 1,266(36.61) | | 1,243 (35.95) | | 0.0138 | |  |
| **ARBs/ACEIs** | 279 (3.38) | | 288 (3.49) | | 0.0060 | | 149 (4.31) | | 147 (4.25) | | 0.0029 | |  |
| **Statins** | 1903 (23.06) | | 1917 (23.23) | | 0.0040 | | 702 (20.30) | | 683 (19.75) | | 0.0137 | |  |
| **Anti-diabetes drugs** | 2162 (26.20) | | 2171 (26.31) | | 0.0025 | | 867 (25.07) | | 857 (24.78) | | 0.0067 | |  |
| ^a^ Data was expressed as mean ± standard deviation or number (percentage) unless specified otherwise. The co-morbid diseases were identified from diagnoses within 1 year before the first date of NOAC prescription.  ^b^ CHA_2_DS_2_-VASc scores ranged from 0 to 9; a higher score indicates a higher risk of stroke or thromboembolism. One point was assigned for congestive heart failure, hypertension, age between 65-74 years, diabetes mellitus, and vascular disease. Two points were assigned for age ≥ 75 years, previous stroke, transient ischemic attack, and systemic thromboembolism.  ^c^ HAS-BLED scores ranged from 0 to 9; a higher score indicating a higher risk for major bleeding. One point was assigned for hypertension, renal disease, liver disease, stroke, bleeding, age > 65 years, treatment with platelet inhibitors or non-steroidal anti-inflammatory drugs, and alcohol abuse.  **Abbreviations:** ACEIs: angiotensin converting enzyme inhibitors; AMI: acute myocardial infarctions; ARBs: angiotensin receptor II blockers; CCBs: calcium channel blockers; DHP: dihydropyridine; DM: diabetes mellitus; GI: gastrointestinal; H2: histamine receptor 2; ICH: intracranial hemorrhage; NSAIDs: non-steroidal anti-inflammatory drugs; PPIs: proton pump inhibitors; PVD: peripheral vascular disease; STE: systemic thromboembolism; TIA: transient ischemic attack; VTE: venous thromboembolism. | | | | | | | | | | | | |  |
